# Supplementary material for: Classification of very low birth weight infants as small for gestational age: International vs. national standards
Source: PLoS One. 2025 May 8;20(5):e0323470. doi: 10.1371/journal.pone.0323470 (PMC12061126; doi:10.1371/journal.pone.0323470)
Supplement: Attachment — Submitted filename: Response to Reviewers.docx [file pone.0323470.s002.docx]

**REBUTTAL LETTER**

Marta Tejón Fernández, MSc

Foundation for Biosanitary Research in Eastern Andalusia (FIBAO)

Torrecardenas Hospital - Hermandad de Donantes de Sangre Street

04009, Almeria, Spain

[martatejondn@gmail.com](mailto:martatejondn@gmail.com)

December 6th, 2024

Academic Editor and Reviewers

PLOS ONE

PLOS Journals

Dear Editor and Reviewers,

We sincerely appreciate the time and effort you have dedicated to reviewing our manuscript, titled "Small-for-gestational-age in very low birth weight: International vs. national standards”. Your detailed feedback and constructive suggestions have been invaluable in improving the quality and clarity of our work.

In this letter, we provide a point-by-point response to your comments, highlighting the revisions made to address each of your concerns. You will find our responses below your comments, highlighted in blue.

Thank you for your valuable feedback and the opportunity to improve our manuscript.

Yours sincerely,

The authors.

Journal Requirements:

1. When submitting your revision, we need you to address these additional requirements.

Please ensure that your manuscript meets PLOS ONE's style requirements, including those for file naming. The PLOS ONE style templates can be found at https://journals.plos.org/plosone/s/file?id=wjVg/PLOSOne_formatting_sample_main_body.pdf and https://journals.plos.org/plosone/s/file?id=ba62/PLOSOne_formatting_sample_title_authors_affiliations.pdf

The revised manuscript has been prepared in accordance with the journal's style requirements.

2. In the online submission form, you indicated that All data underlying the results presented in the study are available from Marta Tejón-Fernández (martatejondn@gmail.com), who is the first and corresponding author.

All PLOS journals now require all data underlying the findings described in their manuscript to be freely available to other researchers, either a. In a public repository, b. Within the manuscript itself, or c. Uploaded as supplementary information.

This policy applies to all data except where public deposition would breach compliance with the protocol approved by your research ethics board. If your data cannot be made publicly available for ethical or legal reasons (e.g., public availability would compromise patient privacy), please explain your reasons on resubmission and your exemption request will be escalated for approval.

The data have been uploaded to the Figshare repository (http://dx.doi.org/10.6084/m9.figshare.27985910).

1. Is the manuscript technically sound, and do the data support the conclusions?

The manuscript must describe a technically sound piece of scientific research with data that supports the conclusions. Experiments must have been conducted rigorously, with appropriate controls, replication, and sample sizes. The conclusions must be drawn appropriately based on the data presented.

Reviewer #1: Partly

Reviewer #2: Yes

Thank you, reviewers. In response to Reviewer #1’s comment, in the manuscript, we have included comments regarding the limitation related to the sample size. Furthermore, we have undertaken a revision of the conclusions to ensure their alignment with the stated objectives, substantiating them with the results obtained.

2. Has the statistical analysis been performed appropriately and rigorously?

Reviewer #1: Yes

Reviewer #2: Yes

3. Have the authors made all data underlying the findings in their manuscript fully available?

The [PLOS Data policy](http://track.editorialmanager.com/CL0/http:%2F%2Fwww.plosone.org%2Fstatic%2Fpolicies.action%23sharing/1/010f019356452c72-141178b6-bb03-4fa7-abab-d7b08f16473a-000000/IU0Ue_mEx0EH6pI_UXicvmlfSS-g9orfue5f9RgZGCA=186) requires authors to make all data underlying the findings described in their manuscript fully available without restriction, with rare exception (please refer to the Data Availability Statement in the manuscript PDF file). The data should be provided as part of the manuscript or its supporting information, or deposited to a public repository. For example, in addition to summary statistics, the data points behind means, medians and variance measures should be available. If there are restrictions on publicly sharing data—e.g. participant privacy or use of data from a third party—those must be specified.

Reviewer #1: Yes

Reviewer #2: Yes

4. Is the manuscript presented in an intelligible fashion and written in standard English?

PLOS ONE does not copyedit accepted manuscripts, so the language in submitted articles must be clear, correct, and unambiguous. Any typographical or grammatical errors should be corrected at revision, so please note any specific errors here.

Reviewer #1: No

Reviewer #2: No

The manuscript has undergone a comprehensive review to address typographical and grammatical errors. We have improved the manuscript's writing to ensure its adaptation to standard English.

5. Review Comments to the Author

Please use the space provided to explain your answers to the questions above. You may also include additional comments for the author, including concerns about dual publication, research ethics, or publication ethics. (Please upload your review as an attachment if it exceeds 20,000 characters)

**Reviewer #1:** Fernández and co-authors have compared demographic characteristics and outcomes (mortality and several morbidities) in small-for-gestational age (SGA) babies, selected from a preterm low birth weight population of 250 babies born in a single hospital in Spain, according to whether they were classified as SGA according to national or international birth weight distributions. The analysis involved retrieval of electronic patient records and hardcopy files to obtain information on outcomes, then comparison of outcomes between SGA groups defined by the two classifications. Only five years of data, generating a cohort of 250 babies, was used, and perhaps a larger cohort would have allowed for more rigorous testing of whether rarer outcomes such as sepsis differ depending on SGA classification cut-offs. Clarification of the question and discussion of the implications would improve the manuscript. The English usage is hard to understand in places.

Thank you, Reviewer, for your valuable comments. We have included a discussion in the manuscript regarding the limitations of the sample size, along with suggestions for addressing this issue in future research. Additionally, the manuscript has been thoroughly reviewed to correct grammatical errors and ensure its adaptation to standard English.

Specific comments:

a. Title page – better to provide an institutional Email as contact, rather than gmail.

- At present, Tejón-Fernández does not have an institutional Email.

b. Abstract – this needs additional information, e.g. upper and lower gestational age boundary for the cohort (not just lower limit).

- The upper limit of gestational age has been incorporated into the abstract.

“250 neonates weighing <1500 g, born between 26 and 36 weeks of gestation were included.”

c. English usage – needs correction/clarification in places, e.g.

- Spanish not Spaniard in abstract. Corrected.
- Data are all quantitative not qualitative (statistics in methods – just not all continuous/normally distributed). Corrected, the Chi-squared test and Student's t-test were used for variable comparison.
- Abstract: Differences in outcomes are not between growth charts, but between babies classified according to the two different systems/sets of cut-offs. The suggested corrections have been implemented.
- Line 62: use the “less than or equal symbol” to make this clearer. Corrected.
- Line 65 “being 15% of this percent less than…” – very unclear. As suggested by the reviewers, that paragraph is unclear and contributes little to the manuscript; therefore, the comment has been removed.
- Line 123 babies are male or female, not men or women. The text has been corrected.

“Of this cohort, 52.4% were female”.

- Line 128-129 – what do you mean by minor? Different or not? This has been clarified and corrected in the text.

“The average z-scores were compared between the Carrascosa 2008 and INT-21 standards. When using the INT-21 standards compared to Carrascosa 2008 the z-score was statistically significantly higher for weight (−0.77 vs. −0.99, p < 0.001), length (−0.87 vs. −1.21, p < 0.001) and head circumference (−0.38 vs. −1.21, p < 0.001).”

- Line 147 “it also did not observe differences between” – unclear. The text has been revised to enhance clarity.
  “No significant differences were found between neonates classified as SGA using INT-21 and Carrascosa 2008 standards regarding gestational age, birth anthropometry, Apgar scores, NICU stay duration, mechanical ventilation days, parenteral nutrition days, or total hospital stay (p = NS)(Table 1).”

d. Introduction – much of this section was hard to follow. The initial sentences were about prematurity, not classification systems or why this might be important. Grammatical corrections have been made, along with the implementation of the proposed changes.

Lines 57-58 seems a mixed argument comparing VLBW with late preterm not normally grown? The question of classification standards and what has been done previously was not reviewed until the discussion – I recommend reworking the introduction to cover this material and to clearly explain the gap in understanding that the present study addresses. The corrections in the introduction have been made based on the reviewer' suggestions.

Why did you focus on very preterm babies (26-32 weeks) only? There was a transcription error: the preterm neonates in our study are within the range of 26 to 36 weeks of gestation. The choice of 26 weeks was based on the fact that the Carrascosa 2008 standards were established using a cohort starting at 26 weeks of gestation. These details have been corrected and included in the manuscript.

Line 68 – what is EEUU? Define abbreviations. Corrected.

“Regarding SGA neonates, a cohort of infants weighing less than 1500 grams (g) in the United States in 2021…”.

Line 87 – what do you mean by “adequate pregnancy?” It has been clarified in the text.

“The study included low-risk mothers who were nonsmokers, did not use drugs, experienced uncomplicated pregnancies…”.

I think the sentence at lines 72-74 is intended as a statement of the rationale for the present study, but it is very hard to follow. The rationale for the study has been clarified, and one of the bibliographic references has been modified to enhance understanding.

15. Rajendra Prasad A, Venkateshwarlu V, · Srinivas M, Sai Kiran D and· Tejo Pratap O. Comparison of Fenton, INTERGROWTH 21st, and Population Based Growth Charts in Predicting Outcomes of Very Preterm Small for Gestational Age Neonates. Indian Journal of Pediatrics. 2022; 89(10): 1034–36. https://doi.org/10.1007/s12098-022-04175-3 PMID: 35604586

The statement of the aim (lines 90-93) is confusing; could you split this into two parts? The aims of the study have been clarified in two sections.

“The aims of this study were a) to evaluate the concordance between SGA very low birth weight neonates classified using the Spanish preterm growth standard (Carrascosa 2008) and those classified using the international growth standard (INT-21); b) to compare the incidence, morbidity, and mortality rates of SGA neonates in a cohort of Spanish VLBW infants, classified using either the international INT-21 growth standard, which does not include Spanish neonates, or the Spanish preterm growth standard (Carrascosa 2008).”.
e. Methods – do you have access to other important outcomes for this preterm cohort, e.g. need for supplemental oxygen at discharge? Respiratory distress syndrome? Only the days of supplemental oxygen were recorded. Give manufacturer and city in brackets not text – is there also a catalog number for the incubator that was used? The incubator used was the Caleo model, manufactured by Dräger Medical, catalog code 2M50555-23.

f. Ethics – this is usually placed at the start of the methods.

The ethical considerations have been included at the beginning of the Methods section, as suggested by the reviewers.

“Ethics approval for the project was obtained from the Research Ethics Committee of Torrecardenas Hospital (CEI-Almeria code: 9/2017, on March 29th, 2017). Since the study was a retrospective study using anonymized data, individual patient consent was not required. Access to the data was on the 2nd of February 2018.”

g. Results – text should be revised to present data only to meaningful levels of precision. For example, the methods state that the birth weight of babies could be measured to the nearest gram, so weight should be presented as whole grams only. Similarly, if you are measuring head circumference with a tape, the precision is not going to be greater than 0.1 cm at best. The level of precision in the tables is better, although editing is needed to ensure that all means and standard deviations for a given outcome are described with the same number of decimal places. The results have been revised based on the improvement suggestions provided by the reviewers.

h. Figure 1 – I suggest removing Figure 1, because its’ contents are very unclear and it is a poor-quality figure. Instead, you could provide the actual data and z-scores for average weight, crown-rump length and head circumferences for the cohort according to each classification system in a Table. Providing mean plus/minus standard deviation for z-scores would be more informative than only mean. I also recommend that you shift the sentences on gestational age from the end of the results into this first section of results – this data could also be added to an initial “demographics” table, which could include the z-scores for the cohort of babies according to each classification system.

Figure 1 has been removed, and the corresponding results have been specified more precisely in the text.

i. Table 1 is not needed – just place the numbers and percentages in text and avoid repeating these. Table 1 has been removed, and the results are now presented in the text.

j. Tables 2 and 3 – it would be more logical to reorganise these so that demographic information is in one table and all of the outcomes related to morbidity (including NICU admission, supplemental oxygen, BPD, IVH etc) are in the other. The modifications suggested by the reviewers have been implemented.

k. Table 4 and 5 are not relevant to your question and could be removed. Differences between AGA and SGA are well known, not novel and not related to your question about whether outcomes differ according to which classification system is used to define SGA. That question is already addressed in actual Table 2. Tables 4 and 5 have been removed as suggested by the reviewers.

l. Discussion – This section would be improved by rewriting to clearly discuss the findings of the present study and its implications. Descriptions of what others have found when comparing classification systems belongs in the introduction to clearly explain what gap in knowledge you are addressing. These also need to be better integrated to explain the overall argument as this currently reads as a series of paragraphs each describing a single study, which are not clearly linked.
Based on the improvement suggestions and questions raised by the reviewers, we have revised the Discussion section and conducted the following statistical analysis, which has been added to the Results section. This analysis allows us to address most of the questions posed by the reviewers.

“The gestational age of the 23 neonates classified as SGA by Carrascosa 2008 but not by INT-21 was similar to the GA of the non-SGA group according to INT-21 (28.9 ± 1.7 weeks vs. 29.5 ± 2.0 weeks, p = 0.10). However, their birth weight was statistically significantly higher (1217 ± 201 g vs. 1094 ± 238 g, p = 0.004). No differences were observed in mortality or morbidity outcomes between these groups (p = NS).”

The Discussion section has been revised to incorporate the considerations suggested by the reviewers.
m. Conclusions – these are fairly limited.

Can you make recommendations about which classification systems should be used in Spain based on your study? The comment has been incorporated into the Discussion and Conclusions sections.

“Both standards classify preterm infants with comparable neonatal morbidity and mortality, making it reasonable to recommend the use of either Carrascosa 2008 or INT-21 for classifying SGA preterm neonates in Spain.”

Is it better to use the local growth charts and define more babies as SGA or the international standard and define fewer babies as SGA? The comment has been included in the Discussion section.

Does this impact the clinical care or decisions made about theses babies? The comment has been included in the Discussion section.

“When analyzing discrepancies between the Carrascosa-2008 and INT-21 standards (23 neonates classified as SGA by Carrascosa-2008 but not by INT-21), we found that these neonates had lower birth weights but similar gestational ages, morbidity, and mortality compared to non-SGA neonates classified by INT-21.”

Would using local standards limit ability to compare studies internationally? The comment has been included in the Discussion and Conclusions section.

“Given that studies conducted in Spain using INT-21 report SGA prevalence rates in VLBW neonates consistent with those found in international studies, we recommend adopting INT-21 as the preferred growth standard for comparing SGA neonates across studies conducted in different countries.”

Are your findings relevant to other populations, or only in the Spanish context? The comment has been included in the Discussion section.

“Neonatologists often face uncertainty about which growth standard to use for classifying SGA preterm neonates, and there is ongoing debate about whether to use national standards or international ones that encompass diverse populations to better identify preterm at risk for complications.”

**Reviewer #2:** The rationale for the study is justified since Spanish population was not part of the intergrowth 21 data set. Statistical methodology is also acceptable. The authors should consider correcting multiple grammatical errors before resubmission.

Thank you for your comments, Reviewer. The manuscript has been thoroughly reviewed to correct grammatical errors and to improve the English writing, aiming to facilitate its understanding and clarity.

6. PLOS authors have the option to publish the peer review history of their article ([what does this mean?](https://track.editorialmanager.com/CL0/https:%2F%2Fjournals.plos.org%2Fplosone%2Fs%2Feditorial-and-peer-review-process%23loc-peer-review-history/1/010f019356452c72-141178b6-bb03-4fa7-abab-d7b08f16473a-000000/4JSB8-goXhIx6lyhf6bJG99kW2GFc2wc4QMFfN9JC4A=186)). If published, this will include your full peer review and any attached files.

If you choose “no”, your identity will remain anonymous but your review may still be made public.

**Do you want your identity to be public for this peer review?** For information about this choice, including consent withdrawal, please see our [Privacy Policy](https://track.editorialmanager.com/CL0/https:%2F%2Fwww.plos.org%2Fprivacy-policy/1/010f019356452c72-141178b6-bb03-4fa7-abab-d7b08f16473a-000000/-5I4VkUciMkc7xaFdOYv1m_nvdxBAqPaAXCxG2kl_x0=186).

Reviewer #1: No

Reviewer #2: No
