# Supplementary material for: Classification of very low birth weight infants as small for gestational age: International vs. national standards
Source: PLoS One. 2025 May 8;20(5):e0323470. doi: 10.1371/journal.pone.0323470 (PMC12061126; doi:10.1371/journal.pone.0323470)
Supplement: Attachment — Submitted filename: Response_to_Reviewers_auresp_2.docx [file pone.0323470.s003.docx]

**REBUTTAL LETTER**

Marta Tejón Fernández, MSc

Foundation for Biosanitary Research in Eastern Andalusia (FIBAO)

Torrecardenas Hospital - Hermandad de Donantes de Sangre Street

04009, Almeria, Spain

[martatejondn@gmail.com](mailto:martatejondn@gmail.com)

March 3rd, 2025

Academic Editor and Reviewers

PLOS ONE

PLOS Journals

Dear Editor and Reviewers,

We would like to extend our sincerest gratitude for the time and effort you have dedicated to reviewing our manuscript, “*Small-for-gestational-age in very low birth weight: International vs. national standards"*. Your insightful comments and constructive feedback have been invaluable in improving the quality and clarity of our work.

We have carefully addressed all the points raised during the review process. The responses to your comments can be found in blue in this letter, ensuring they are easily identifiable. Each suggestion has been thoughtfully considered, and the modifications have been incorporated into the manuscript. We believe that these revisions have significantly improved the manuscript, and we are deeply appreciative of your expertise and guidance throughout this process.

Once again, thank you for your invaluable contributions to our work. We are honoured to have had the opportunity to benefit from your knowledge and professionalism.

Yours sincerely,

The authors.

We have included M. Martín-González as one of the authors of the manuscript. Given their extensive expertise, they have provided invaluable assistance in restructuring the article based on the requested modifications.

The title of the manuscript has been revised following the recommendation of AJE, the editing service commissioned to refine the English language usage, as they indicated that a correction was necessary.

Author's Responses to Reviewers’ questions:

1. If the authors have adequately addressed your comments raised in a previous round of review and you feel that this manuscript is now acceptable for publication, you may indicate that here to bypass the “Comments to the Author” section, enter your conflict of interest statement in the “Confidential to Editor” section, and submit your "Accept" recommendation.

Reviewer #1: (No Response)

Reviewer #2: (No Response)

2. Is the manuscript technically sound, and do the data support the conclusions?

The manuscript must describe a technically sound piece of scientific research with data that supports the conclusions. Experiments must have been conducted rigorously, with appropriate controls, replication, and sample sizes. The conclusions must be drawn appropriately based on the data presented.

Reviewer #1: Yes

Reviewer #2: Yes

3. Has the statistical analysis been performed appropriately and rigorously?

Reviewer #1: Yes

Reviewer #2: Yes

4. Have the authors made all data underlying the findings in their manuscript fully available?

The PLOS Data policy requires authors to make all data underlying the findings described in their manuscript fully available without restriction, with rare exception (please refer to the Data Availability Statement in the manuscript PDF file). The data should be provided as part of the manuscript or its supporting information, or deposited to a public repository. For example, in addition to summary statistics, the data points behind means, medians and variance measures should be available. If there are restrictions on publicly sharing data—e.g. participant privacy or use of data from a third party—those must be specified.

Reviewer #1: Yes

Reviewer #2: Yes

5. Is the manuscript presented in an intelligible fashion and written in standard English?

PLOS ONE does not copyedit accepted manuscripts, so the language in submitted articles must be clear, correct, and unambiguous. Any typographical or grammatical errors should be corrected at revision, so please note any specific errors here.

Reviewer #1: No

Reviewer #2: No

The language-related corrections have been carried out by a professional editing company, AJE, which has revised and edited the text to ensure its adherence to the appropriate linguistic standards. The certification is included in the documents submitted to the journal.

6. Review Comments to the Author

Please use the space provided to explain your answers to the questions above. You may also include additional comments for the author, including concerns about dual publication, research ethics, or publication ethics. (Please upload your review as an attachment if it exceeds 20,000 characters)

Reviewer #1: Thank you for addressing many of the comments made on the original manuscript, including clarification of the aims statement and substantial revision of the results Tables. There are some areas where I think the manuscript could still be improved, and/or further changes are needed to appropriately address the comments. In some cases the information was provided in responses but not incorporated into the actual manuscript. Please see points below. Could you please indicate the page and line of the tracked changes version where further changes are made, to make re-review faster?

Thank you very much for all your contributions. Although some paragraphs have been removed and others have been substantially modified, In response to your request, we have enabled line numbers in the track changes document. This will allow you to view all modifications made to the manuscript, along with their corresponding justifications.

• The English usage/wording is still unclear in places, e.g. first sentence of abstract (not a complete sentence and meaning is unclear): “The comparability of the Spanish neonatal growth standards (Carrascosa 2008) and the international INTERGROWTH-21 standards in classifying very low birth weight neonates with the same morbidity and mortality rates as small-for-gestational-age is unknown, and what the degree of concordance between these standards is.”

The English usage/wording has been reviewed by AJE, this sentence has been completely modified.

Lines 34-39 (page 2/18): “It is not precisely known whether the use of national (Carrascosa 2008) and international growth standards (INTERGROWTH-21) shows good concordance in classifying very low birth weight infants as small-for-gestational-age or whether with the same degree of morbidity and mortality.”

• I also suggest reworking the introduction further to clearly introduce the question of the present study. It is not clear why the introduction starts with a discussion of VLBW and then a section on incidence on preterm birth, when the question this study addresses is about SGA classification and impact. The introduction relevant to this study is not stated until much later (sentence starting “classifying the growth status”). A point for the authors to note is that not all SGA are preterm.

Revisions have been made to the text in accordance with the request, ensuring a clear introduction of the research question (page 3-5/18).

• The rationale for using a cohort between 26 and 36 weeks should be added to the manuscript.

The justification has been added to the Methods section.

Lines 132-134 (page 6/18): “The inclusion criteria were VLBW neonates with a gestational age between 26 and 36 weeks (since the Carrascosa 2008 standards do not apply to newborns under 26 weeks of gestation).”

• Incubator model and manufacturer location that were provided in the response need to be incorporated in the manuscript.

You are right. We apologize for the oversight, as we did not include the incubator model in the manuscript during the previous revision.

Lines 141-142 (page 6/18): “Weight was measured using the precision scale integrated into the incubator (Caleo model, manufactured by Dräger Medical, Germany; catalog code 2M50555-23)…”

• Thank you for revising the Tables. The point about precision of results, and ensuring means and errors are reported to the same number of decimal places needs further attention, as this has been missed in places, e.g. line 187 in tracked changes manuscript.

The correction has been made, keeping one decimal place for height and head circumference, as you suggested.

Lines 158-160 (page 7/18): “The mean gestational age of the neonates was 29.69 ± 2.2 weeks, with a mean birth weight of 1162.3 ± 235.8 g, a mean length of 37.4 ± 2.8 cm, and a mean head circumference of 26.8 ± 2.1 cm.”

• The point added at line 271 (Neonatologists often face uncertainty about which growth standard to use for classifying SGA preterm neonates, and there is ongoing debate about whether to use national standards or international ones that encompass diverse populations to better identify preterm at risk for complications.”) provides good rationale for the present study and would work well in the introduction), but does not provide a conclusion or recommendation based on the findings of this study and of previous literature, which would be expected in a discussion section. It also does not address the reviewer query: “Are your findings relevant to other populations, or only in the Spanish context?”! Can the findings of THIS study be generalised to other populations, or are they only important for classifying Spanish babies?

We have addressed your valuable comments in the discussion section, further elaborating on the justification for the study. Additionally, we have refined the conclusions to reflect the insightful points raised during the review process (page 9-12/18).

Reviewer #2: Authors have diligently addressed the points raised in the rebuttal letter.

We greatly appreciate your comments. As requested by Reviewer #1, we have included line numbers and page in the track changes document to facilitate your re-review process.

However, a few improvements are necessary to bring the submission to an acceptable standard:

• Editing the grammatical errors in lines 31-32 and 66-70 will enhance readability.

The grammatical errors highlighted have been corrected and reviewed by AJE, the editing service we commissioned to enhance the English language usage. We have attached the certification provided by AJE for the journal's reference.

• The claim of accessing data in just one day (Feb 2nd, 2018) seems exceptionally efficient, almost reaching implausible limits. It may be beneficial to either provide a plausible range or leave it unchanged if accurate.

Access to the data was granted for a period of two months, this has been included in the manuscript.

Lines 126-129 (page 5-6/18): “Access to the data occurred from the 2nd of February 2018 to the 1st of April 2018.”

• The inclusion criteria for INT-21 were based on maternal characteristics (lines 85-87) and differ from those chosen by the authors (lines 110-112). While the patient populations may end up similar in practical terms, an explanation for this deviation is essential. This deviation could potentially explain the differences in outcomes.

The comment has been incorporated into the Discussion section.

Lines 198-202 (page 9/18): “In countries that use the INT-21 standard as the growth standard, the rates of small-of-gestational-age among very low birth weight neonates range from 25% to 35% [1,4,12,14]. The differences in the incidence of SGA among different studies may occur due to the inclusion of different populations of pregnant women. In our study, we did not exclude pregnant women with gestational diabetes or eclampsia, whereas these conditions were excluded in the INT-21 study [5].”

• The Z scores for weight, length, and head circumference show statistically significant differences, with a substantial observed difference of an additional 23 neonates classified as SGA using Carrascosa 2008. Although I concur with the calculated concordance using Cohen’s kappa statistic, providing additional statistical rationale could enhance reader acceptance.

The Discussion now includes the finding that the agreement rate for classifying SGA according to the two standards is 91.8%, which underscores the high level of concordance. However, all discrepancies are attributed to 23 newborns, all of whom were classified as SGA according to Carrascosa 2008 but none according to INT-21. This accounts for the differences in the incidence rates of SGA.

7. PLOS authors have the option to publish the peer review history of their article (what does this mean?). If published, this will include your full peer review and any attached files.

If you choose “no”, your identity will remain anonymous but your review may still be made public.

Do you want your identity to be public for this peer review? For information about this choice, including consent withdrawal, please see our Privacy Policy.

Reviewer #1: No

Reviewer #2: No
