# Supplementary material for: Classification of very low birth weight infants as small for gestational age: International vs. national standards
Source: PLoS One. 2025 May 8;20(5):e0323470. doi: 10.1371/journal.pone.0323470 (PMC12061126; doi:10.1371/journal.pone.0323470)
Supplement: Attachment — Submitted filename: Response_to_Reviewers_auresp_3.docx [file pone.0323470.s004.docx]

**REBUTTAL LETTER**

Marta Tejón Fernández, MSc

Foundation for Biosanitary Research in Eastern Andalusia (FIBAO)

Torrecardenas Hospital - Hermandad de Donantes de Sangre Street

04009, Almeria, Spain

[martatejondn@gmail.com](mailto:martatejondn@gmail.com)

April 2st, 2025

Academic Editor and Reviewers

PLOS ONE

PLOS Journals

Dear Editor and Reviewers,

We sincerely appreciate the time and effort you have devoted to reviewing our manuscript, *"Classification of very low birth weight infants as small for gestational age: International vs. national standards."*. Your thoughtful feedback and valuable insights have been instrumental in enhancing the clarity and overall quality of our work.

In this revision, we have carefully addressed the minor points raised. For clarity, our responses to your comments are provided in blue within this letter. All suggested revisions have been carefully considered, and the necessary modifications have been implemented in the manuscript.

We are truly grateful for your expertise and guidance throughout this process. Your constructive input has been invaluable in strengthening our study, and we greatly appreciate the opportunity to refine our work under your review.

Yours sincerely,

The authors.

**Journal Requirements:**

Please review your reference list to ensure that it is complete and correct. If you have cited papers that have been retracted, please include the rationale for doing so in the manuscript text, or remove these references and replace them with relevant current references. Any changes to the reference list should be mentioned in the rebuttal letter that accompanies your revised manuscript. If you need to cite a retracted article, indicate the article’s retracted status in the References list and also include a citation and full reference for the retraction notice.

All references have been thoroughly reviewed, both for style and validity. To the best of our knowledge, no retracted articles have been included. One article with an erratum is cited, which is noted in the reference itself.

**Additional Editor Comments:**

Dear Authors,

please make few minor changes regarding text style, requested by the reviewers. After their implementation, your manuscript can be accepted for the publication.

We sincerely appreciate the opportunity for our manuscript to be considered for publication in your esteemed journal.


Reviewers' comments:

Reviewer's Responses to Questions

**Comments to the Author**

1. If the authors have adequately addressed your comments raised in a previous round of review and you feel that this manuscript is now acceptable for publication, you may indicate that here to bypass the “Comments to the Author” section, enter your conflict of interest statement in the “Confidential to Editor” section, and submit your "Accept" recommendation.

Reviewer #1: All comments have been addressed

Reviewer #2: (No Response)

2. Is the manuscript technically sound, and do the data support the conclusions?

The manuscript must describe a technically sound piece of scientific research with data that supports the conclusions. Experiments must have been conducted rigorously, with appropriate controls, replication, and sample sizes. The conclusions must be drawn appropriately based on the data presented.

Reviewer #1: (No Response)

Reviewer #2: Yes

3. Has the statistical analysis been performed appropriately and rigorously?

Reviewer #1: (No Response)

Reviewer #2: Yes

4. Have the authors made all data underlying the findings in their manuscript fully available?

The [PLOS Data policy](http://track.editorialmanager.com/CL0/http:%2F%2Fwww.plosone.org%2Fstatic%2Fpolicies.action%23sharing/1/010f0195f051f29d-b7d33ebb-befa-4e87-a9dc-f018a8858d86-000000/iTef9wXIjBqU8H_OSraMY80-lSoFOGrrHFuetMNq818=204) requires authors to make all data underlying the findings described in their manuscript fully available without restriction, with rare exception (please refer to the Data Availability Statement in the manuscript PDF file). The data should be provided as part of the manuscript or its supporting information, or deposited to a public repository. For example, in addition to summary statistics, the data points behind means, medians and variance measures should be available. If there are restrictions on publicly sharing data—e.g. participant privacy or use of data from a third party—those must be specified.

Reviewer #1: (No Response)

Reviewer #2: Yes

5. Is the manuscript presented in an intelligible fashion and written in standard English?

PLOS ONE does not copyedit accepted manuscripts, so the language in submitted articles must be clear, correct, and unambiguous. Any typographical or grammatical errors should be corrected at revision, so please note any specific errors here.

Reviewer #1: (No Response)

Reviewer #2: Yes

6. Review Comments to the Author

Please use the space provided to explain your answers to the questions above. You may also include additional comments for the author, including concerns about dual publication, research ethics, or publication ethics. (Please upload your review as an attachment if it exceeds 20,000 characters)

**Reviewer #1:** (No Response)

**Reviewer #2:** The authors have taken good efforts to address most of the issues. Reasoning behind results have been included in the discussion. I agree that not excluding preeclamptic mothers can contribute to increased ratio of SGA infants (depending on maternal ratio, of course). Simple, minor changes will enhance readability further.
Line 79: "of Caucasian" can be changed to "Caucasian" or "of Caucasian origin".

This sentence has been modified in accordance with your suggestion.

“…whose parents were Caucasian and born in Spain”

Line 109: "and" can be omitted between length, head circumference.

We have corrected this sentence based on the feedback provided.

“The following variables were analyzed: gestational age, birth weight, length, head circumference, Apgar score, bronchopulmonary dysplasia (BPD)…”

Line 112: "," can be added after total hospital days (in days).

This line has been revised according to your comment.

“…sepsis, total NICU stay (in days), days of mechanical ventilation, total hospital stay (in days), and mortality.”

7. PLOS authors have the option to publish the peer review history of their article ([what does this mean?](https://track.editorialmanager.com/CL0/https:%2F%2Fjournals.plos.org%2Fplosone%2Fs%2Feditorial-and-peer-review-process%23loc-peer-review-history/1/010f0195f051f29d-b7d33ebb-befa-4e87-a9dc-f018a8858d86-000000/H7Fd8gM1Yfzke-Ssg292SsjRZDTP6EEG1DF-5DWgVr4=204)). If published, this will include your full peer review and any attached files.

If you choose “no”, your identity will remain anonymous but your review may still be made public.

**Do you want your identity to be public for this peer review?** For information about this choice, including consent withdrawal, please see our [Privacy Policy](https://track.editorialmanager.com/CL0/https:%2F%2Fwww.plos.org%2Fprivacy-policy/1/010f0195f051f29d-b7d33ebb-befa-4e87-a9dc-f018a8858d86-000000/zru4A0xJzcvvetrgToqZe9hmR0PZwM0gKHBxJrM2xaM=204).

Reviewer #1: No

Reviewer #2: No
